# Impaired beta cell function is present in nondiabetic rheumatoid arthritis patients

**DOI:** 10.1186/ar4149

**Published:** 2013-01-22

**Authors:** Iván Ferraz-Amaro, Jose A García-Dopico, Lilian Medina-Vega, Miguel A González-Gay, Federico Díaz-González

**Affiliations:** 1 Division of Rheumatology, Hospital Universitario de Canarias, Ofra, s/n, La Laguna 38320, Santa Cruz de Tenerife, Spain; 2 Servicio de Laboratorio, Hospital Universitario de Canarias, Ofra, s/n, La Laguna 38320, Santa Cruz de Tenerife, Spain; 3 Division of Rheumatology, Hospital Universitario Marqués de Valdecilla, IFIMAV, Avenida Valdecilla, s/n, 39008 Santander, Cantabria, Spain; 4 Departamento de Medicina, Facultad de Medicina, University of La Laguna, Ofra, s/n. La Laguna, 38320 Santa Cruz de Tenerife, Spain

## Abstract

**Introduction:**

To investigate how markers of β-cell secretion (proinsulin-processing metabolites) are expressed in rheumatoid arthritis (RA) patients and their potential relation with the insulin resistance (IR) observed in these patients.

**Methods:**

The 101 RA patients and 99 nondiabetic sex- and age-matched controls were included. IR by homeostatic model assessment (HOMA2), and β-cell secretion, as measured by insulin, split and intact proinsulin, and C-peptide levels were determined for both groups. Multiple regression analysis was performed to compare IR between groups and to explore the interrelations between RA features, proinsulin metabolites, and IR. Data were adjusted for glucocorticoids intake and for IR classic risk factors.

**Results:**

Compared with controls, RA patients showed higher HOMA-IR (β coef., 0.40 (95% CI, 0.20 to 0.59); *P *= 0.00). When data were adjusted for glucocorticoids intake, noncorticosteroid patients maintained a higher IR index (β, 0.14 (0.05 to 0.24); *P *= 0.00). Impaired insulin processing in RA patients was detected by the onset of elevated split proinsulin levels (β, 0.70 pmol/L (0.38 to 1.02); *P *= 0.00). These data remained significant also when adjusted for prednisone intake (β, 0.19 (0.00 to 0.36) pmol/L; *P *= 0.04). Split proinsulin-to-C-peptide ratios were higher in RA patients undergoing corticosteroid therapy (β, 0.25 (0.12 to 0.38); *P *= 0.03) and were nearly significant in comparison between noncorticosteroids patients and controls (β, 0.16 (-0.02 to 0.34); *P *= 0.08). Interestingly, the impact of HOMA-IR on the ratio of intact proinsulin to C-peptide was higher in controls compared with patients (β, 6.23 (1.41 to 11.06) versus 0.43 (-0.86 to 1.71); *P *= 0.03).

**Conclusions:**

β-Cell function is impaired in nondiabetic and in RA patients not taking corticoids by a mechanism that seems to be, at least in part, independent of IR.

## Introduction

Rheumatoid arthritis (RA) is a chronic, systemic, inflammatory disorder of unknown etiology that, if left uncontrolled, may lead to the destruction and deformity of joints because of the erosion of cartilage and bone. Although a higher prevalence of traditional cardiovascular risk factors may be present in patients with RA than in the general population, epidemiologic data suggest that RA is an independent risk factor for cardiovascular disease and may be a major contributor to increased patient mortality [1,2].

Several reports have linked clinical RA activity with a reduction in peripheral insulin sensitivity [3-7]. Insulin resistance (IR) refers to a state in which a given concentration of insulin is associated with a subnormal glucose response [8]. IR is the primary defect underlying the development of type 2 diabetes mellitus and is a key component defining the metabolic syndrome, a constellation of abnormalities including obesity, hypertension, glucose intolerance, and dyslipidemia that may eventually lead to cardiovascular disease. As IR has recently been associated with states of low-grade inflammation, it is now believed that inflammation might contribute to its development [9]. Similarly, IR is now additionally recognized as a component of several disorders in which a chronic inflammatory state is present, such as RA [10].

A complex network of inflammatory cytokines, adipocytokines, transcription factors, receptor molecules, and acute-phase reactants are involved in the development of IR. Both peripheral insulin action and insulin secretion have been shown to be impaired in IR states, leading to an increased proportion of insulin precursor, proinsulin [11]. These findings are supported by the fact that intermediate proinsulin products like des-31,32 or des-62,64 proinsulin are elevated in IR and diabetes patients [12,13], showing that the processing of proinsulin to insulin in β cells is impaired.

Previous studies that have examined insulin sensitivity and β-cell function in RA have relied solely on fasting parameters, such as HOMA (Homeostatic Model Assessment)-IR and HOMA%B. Although these model-derived indices have been well validated, they provide no information about proinsulin processing or insulin secretion by β cells. In this regard, disproportionate hyperproinsulinemia as an indicator of β-cell dysfunction has not been explored in RA. The aim of this study was to investigate β-cell function (secretion) in RA patients and its potential relation with the IR observed in these patients.

## Materials and methods

### Study participant

Two hundred subjects, 101 RA patients and 99 age- and sex-matched controls, were recruited for this cross-sectional study. These RA patients, men and women ages 18 or older, were diagnosed by a rheumatologist as having fulfilled 2010 ACR/EULAR diagnostic criteria [14]. For inclusion in the present study, RA disease duration was required to be ≥ 1 year. To minimize the potential effects of tumor necrosis factor (TNF) blockers on IR [5], RA patients undergoing TNF-antagonist therapy were not included in the present study. However, because corticosteroids are often used in the management of RA, patients taking prednisone or an equivalent dose (12 mg/day or less) were not excluded. Nevertheless, we established two groups within the cohort of RA patients; those receiving prednisone therapy, which included subjects then being treated with such drugs or who had been taking corticosteroids within 3 months before the onset of the study, and those not taking corticosteroids, which included RA patients who had not received corticosteroids during the 12 months before recruitment. The glucocorticoid dose was measured as the equivalent prednisone dose administered during the previous 3 months (milligrams per day). Patients and controls with diabetes mellitus were not included in the study. Therefore, none of the patients or controls was receiving glucose-lowering drugs or insulin therapy. All patients and controls had a glucemia < 7 mmol/L. Patients and controls were excluded if they had a history of myocardial infarction, angina, stroke, a glomerular filtration rate < 60 ml/min/1.73 m^2^, a history of cancer, or any other chronic disease, or evidence of infection. None of the controls was receiving corticosteroid treatment. The study protocol was approved by the institutional review committee at Hospital Universitario de Canarias (Spain), and all subjects provided written informed consent.

## Data collection

Patient surveys for both the RA and control groups were identical, except that subjects with RA were asked additional questions pertaining to their disease. Subjects completed a cardiovascular risk factor and medication use questionnaire and underwent a physical examination to assess their anthropometrics and blood pressure. Medical records were reviewed to ascertain the specific diagnosis and medication. Weight was assessed to the nearest 100 g with the participant standing on a portable digital scale (SECA, Hamburg, Germany). Standing height was measured to the nearest 1 cm with a stadiometer (SECA). Waist circumference was measured at the smallest circumference between the rib cage and the iliac crest, while the subject was in a standing position. The hip circumference was measured at the widest circumference between the waist and thighs. The waist-to-hip ratio also was estimated. In patients with RA, disease activity was measured by using the Disease Activity Score (DAS28) in 28 joints [15], whereas disease disability was determined by using the Health Assessment Questionnaire (HAQ) [16]. The metabolic syndrome was defined based on the 2005 National Cholesterol Education Program (Adult Treatment Panel (ATP) III) criteria) [17].

### Assessments

The homeostatic model assessment (HOMA) method was performed to determine IR; specifically, in this study, we used HOMA2: the updated-computer HOMA model [18-20]. In brief, this method consists of a structural computer model of the glucose-insulin feedback system in a homeostatic (overnight-fasted) state. The model comprises a number of nonlinear empirical equations (and precludes an exact algebraic solution), which describes the functions of organs and tissues involved in glucose regulation. This model can be used to determine insulin sensitivity (%S) and β-cell function (%B) from paired fasting plasma glucose and specific insulin, or C-peptide concentrations across a range of 1 to 2,200 pmol/L for insulin and 1 to 25 mmol/L for glucose. In our study, we used C-peptide to calculate β-cell function, because the former is a marker of secretion. In addition, we used insulin data to calculate %S (because HOMA-%S is derived from glucose disposal as a function of insulin concentration). This computer model provides an insulin-sensitivity value expressed as HOMA2-%S (where 100% is normal). HOMA2-IR (insulin resistance index) is simply the reciprocal of %S.

Insulin (Architect Abbott, 2000I) and C-peptide (Immulite 2000, Siemens) were determined with chemiluminescent immunometric assays. Intact proinsulin was assessed through ELISA (Millipore, Billerica, MA, USA). This ELISA does not cross-react with human insulin, and the presence of insulin (up to 208 μU/ml) in serum does not interfere with the assay results. The kit has no cross-reactivity with the major species of proinsulin metabolites, des(31,32) proinsulin, and cross-reacts only weakly with the minor intermediate des(64,65) proinsulin (interassay precision, 3.3% to 9.0%; intraassay, 0.4% to 7.7%). Total (split) proinsulin was detected with ELISA (Millipore). This kit has 100% cross-reactivity with intact human proinsulin and its major processed intermediate, des(31,32) proinsulin, and 81% cross reactivity with its processed intermediate des(64,65) proinsulin. Human insulin (up to 200 μU/ml) and human C-peptide (up to 10 ng/ml) do not interfere with the assay results. Precision was estimated as interassay, 2.9% to 8.3%; intraassay, 0.8% to 8.5%. A split proinsulin to C-peptide ratio was used as a surrogate marker of β-cell function [21]. Standard techniques were used to measure plasma glucose, C-reactive protein (CRP), the Westergren erythrocyte sedimentation rate (ESR), and serum lipids.

### Statistical analysis

On the basis of previously published findings [13,21], we assumed a normal intact proinsulin to C-peptide ratio of 10% in controls and 14% in patients with insulin. Proceeding with these assumptions, by using a 1:1 relation, and according to a Student *t *test with an α level of 0.05 and a β level of 0.15, we estimated that we would need to enroll 184 subjects: 92 patients and 92 controls. Demographic and clinical characteristics shown in Table 1 were compared between patients with RA and controls by using χ^2 ^tests for categoric variables or Student *t *tests for continuous variables (data described as mean ± standard deviation). For noncontinuous variables, either a Mann-Whitney *U *test was performed, or a logarithmic transformation was made, and data were expressed as a median (interquartile range). Association of insulin sensitivity and β-cell function with clinical features of RA, as well as comparisons between RA patients and controls, were conducted through multivariate analysis, adjusting for factors known to be associated with IR. Two different models were defined for the results obtained from these analyses. An unadjusted model in which RA features and metabolic syndrome factors were associated through univariate analysis, and an adjusted model in which RA features were correlated with insulin-sensitivity parameters after adjustment for these classic risk factors through multiple regression also were carried out. A dissimilar impact of HOMA-IR on proinsulin metabolites in patients and controls was established, adding an interaction term, by using multivariate regression analysis. All analyses used a 5% two-sided significance level and were performed by using SPSS software, version 19 (IBM, Chicago, IL, USA). A *P *value < 0.05 was considered statistically significant.

**Table 1:** Demographics and disease-related characteristics of patients and controls

	**Rheumatoid arthritis (*n *= 101)**				**Controls (*n *= 99)**	***P***^a^
	
		**GC-RA (*n *= 46)**	**GC+RA (55)**	***P***^**b**^		
Age, years	55.2 ± 10.0	53.0 ± 10.1	57.1 ± 9.7	0.04^a^	55.1 ± 10.6	0.90
Female, *n *(%)	86 (85)	38 (67)	48 (87)	0.51	90 (91)	0.21
Body mass index, kg/m^2^	29.5 ± 5.9	27.8 ± 4.8	30.0 ± 6.6	0.18	29.5 ± 5.4	0.59
Hip circumference, cm	106 (99-118)	105 (97-115)	109 (99-118)	0.17	102 (98-110)	0.23
Waist circumference male, cm	106 ± 17	105 ± 12	107 ± 22	0.79	100 ± 12	0.57
Waist circumference female, cm	96 ± 15	92 ± 14	98 ± 15	0.13	91 ± 11	0.12
Waist-to-hip ratio	0.90 (0.85-0.94)	0.89 (0.83-0.94)	0.90 (0.86-0.94)	0.34	0.86 (0.80-0.92)	0.71
Hypertension, *n *(%)	36 (38)	14 (25)	22 (40)	0.51	31 (31)	0.34
Statins intake, *n *(%)	29 (30)	13 (23)	16 (29)	0.78	28 (28)	0.77
Metabolic syndrome, *n *(%)	32 (37)	15 (27)	17 (31)	0.96	18 (18)	0.11
ESR, mm/h	25 (16-39)	25 (15-41)	26 (18-39)	0.70	18 (13-25)	0.00^a^
CRP, mg/dl	3.8 (1.4-9.4)	4.5 (1.3-13.7)	3.2 (1.6-9.0)	0.59	2.0 (0.9-4.7)	0.00^a^
Cholesterol, mg/dl	210 ± 40	203 ± 30	216 ± 46	0.18	207 ± 39	0.62
Triglycerides, mg/dl	118 (85-151)	110 (81-139)	121 (91-152)	0.28	102 (69-133)	0.00^a^
HDL cholesterol, mg/dl	59 ± 15	58 ± 14	60 ± 16	0.48	56 ± 14	0.13
LDL cholesterol, mg/dl	127 ± 33	127 ± 28	127 ± 37	0.89	129 ± 34	0.62
Disease duration, years	7.5 ± 5.8	8.7 ± 4.5	6.2 ± 6.8	0.08		
DAS28-ESR	3.99 ± 1.41	4.11 ± 1.56	3.18 ± 1.27	0.45		
HAQ	0.61 (0.24-1.28)	0.69 (0.13-1.37)	0.55 (0.2-1.23)	0.92		
Positive rheumatoid factor, *n *(%)	64 (65)	21 (38)	43 (78)	0.00^a^		
Current nonbiologic DMARD, *n *(%)	88 (87)	41 (73)	47 (85)	0.58		
Current prednisone, *n *(%)	55 (54.5)					
Prednisone, average mg/day/3 months	6.24 ± 2.54					

^a^Comparison between controls and patients; *P *value < 0.05. **^b^**Comparison between not-taking-corticoids and taking-corticoids patients. Data expressed as mean (± standard deviation) or median (interquartile range). Dichotomous variables are expressed as *n *and percentage. CRP, C-reactive protein; DAS28, Disease Activity Score; DMARD, disease-modifying antirheumatic drug; ESR, erythrocyte sedimentation rate; GC-RA, RA patients not taking corticosteroids; GC+RA, RA patients taking prednisone. HAQ, Health Assessment Questionnaire; HDL, high-density lipoprotein; LDL, low-density lipoprotein.

## Results

### Characteristics of the participants

A total of 200 participants, 101 RA patients and 99 controls, with a mean (± standard deviation) age of 55.2 ± 10.0 years and 55.1 ± 10.6 years (*P *= 0.90), respectively, were included in this study. The demographic and disease-related characteristics of the participants are shown in Table 1. Patients in our series had moderate-active disease as shown by DAS28 (3.99 ± 1.41). Half (54.5%) were taking prednisone (an average dose of 6.2 ± 2.5 mg/day during the last year). No differences were found between patients and controls in regard to BMI, waist circumference, hypertension, or metabolic syndrome. As expected, analyses of ESR and CRP values revealed statistically significant differences between controls and patients. Apart from an increased frequency of positive rheumatoid factor among RA patients receiving corticosteroid therapy, compared with those not taking corticosteroids (78% versus 38%; *P *= 0.00), no significant differences between the two subgroups of RA patients were observed in terms of disease duration, activity scores, age, or BMI.

### Insulin-resistance indices

Insulin-resistance indices are shown in Table 2. RA patients had a higher HOMA-IR index (*log *β coefficient, 0.40 (95% CI, 0.20 to 0.59); *P *= 0.00) and elevated β-cell function, HOMA%B- (*log *β coef. 22% (95% CI, 5 to 39); *P *= 0.01) with respect to controls. When RA patients were divided into prednisone intake or non-intake groups, non-corticoids RA patients showed a statistically significantly higher rate of IR compared with controls (*log *β coef. 0.14 (0.05 to 0.24); *P *= 0.00). Similar results were found with regard to β-cell function, with an elevated HOMA%B in noncorticoid patients when compared with controls (*log *β coef. 11% (95% CI, 2 to 19); *P *= 0.02).

**Table 2:** Association of classic risk factors and RA characteristics with insulin sensitivity and β-cell function in patients and controls

	RA and controls (*n *= 200)		RA patients (*n *= 101)			
			Unadjusted model		Adjusted model	
	
	β coefficient (95% CI)	*P*	β coefficient (95% CI)	*P*	β coefficient (95% CI)	*P*
Insulin resistance (*log*HOMA-IR)						
RA versus controls (β 95% CI for RA, 101, and controls, 99)	**0.40 (0.20-0.59)**	**0.00**				
GC-RA versus controls (β 95% CI for RA, 56, and controls, 99)	**0.14 (0.05-0.24)**	**0.00**				
GC+RA versus controls (β 95% CI for RA, 55, and controls, 99)	**0.17 (0.10-0.24)**	**0.00**				
RA features						
						
Disease duration			-0.02 (-0.06-0.01)	0.19	-0.01 (-0.05-0.03)	0.59
RF positivity			0.00 (-0.39-0.39)	0.99	-0.15 (-0.54-0.24)	0.86
ESR			-0.00 (-0.01-0.01)	0.66	-0.01 (-0.02-0.00)	0.26
CRP			-0.01 (-0.02-0.00)	0.05	-0.01 (-0.02-0.00)	0.07
Current prednisone			0.23 (-0.14-0.60)	0.22	0.04 (-0.32-0.40)	0.81
Prednisone, mg/day/previous 3 months			0.01 (-0.18-0.20)	0.94	-0.23 (-0.46-0.01)	0.47
HAQ			-0.01 (-0.30-0.29)	0.97	0.12 (-0.18-0.41)	0.44
DAS28			-0.00 (-0.14-0.13)	0.95	-0.05 (-0.17-0.07)	0.40
Current nonbiologic DMARD			-0.28 (-1.35-0.79)	0.60	0.08 (-0.67-0.82)	0.83
Classic IR risk factors						
						
Sex			-0.24 (-0.76-0.27)	0.35		
Age			0.00 (-0.02-0.02)	0.83		
BMI			**0.06 (0.03-0.10)**	**0.0**^a^		
Waist circumference			**0.02 (0.01-0.03)**	**0.00**^a^		
Triglycerides			0.00 (0.00-0.01)	0.07		
Hypertension			**0.48 (0.11-0.86)**	**0.01**^a^		
β-cell function (*log*HOMA%B)						

RA versus controls (β 95% CI for RA, 101, and controls, 99)	**22 (5-39)**	**0.01**				
GC-RA versus controls (β 95% CI for RA, 56, and controls, 99)	**11 (2-19)**	**0.02**				
GC+RA versus controls (β 95% CI for RA, 55, and controls, 99)	8 (0-15)	0.05				
RA features						
						
Disease duration			2 (-1-4)	0.27	1 (-2-4)	0.40
RF positivity			10 (21-42)	0.52	11 (-19-41)	0.46
ESR			0 (-1-1)	0.52	0 (-1-1)	0.61
CRP			1 (-2-1)	0.32	0 (-1-1)	0.40
Current prednisone			1 (-28-31)	0.93	3 (-26-32)	0.85
Prednisone, mg/day/previous 3 months			2 (-15-18)	0.83	10 (-8-21)	0.36
HAQ			-3 (-27-21)	0.83	3 (-20-26)	0.81
DAS28			-1 (-12-10)	0.81	-0 (-11-10)	0.95
Current nonbiologic DMARD			-16 (110-79)	0.74	-15 (-100-71)	0.73
Classic IR risk factors						
						
Sex			-52 (-92--12)	**0.0**^a^		
Age			-1 (-3-0)	0.06		
BMI			2 (-1-5)	0.16		
Waist circumference			0 (-1-1)	0.67		
Triglycerides			0 (-0-0)	0.90		
Hypertension			10 (-21-41)	0.53		

HOMA-IR and HOMA%B are considered dependent variables and were log transformed. β-coefficients are expressed log transformed. Β-coefficient refers to changes in insulin resistance (IR) or β-cell function (%B) outcome per unit increase in the indicated characteristic. Sex refers to the change from female to male; current prednisone to the change from not taking corticoids to taking corticosteroids treatment, and RF positivity, to the change from negative to positive rheumatoid factor. Adjusted model represents a model in which RA features are adjusted for the classic risk factors of the previous model with *P *< 20 (BMI, and hypertension in the IR model; and sex, age, and BMI for the %B model). See Table 1 for abbreviations and units. Significant associations (*P *< 0.05) are depicted in bold

As is evident in Table 2, in the multiple regression analysis, none of the IR indices correlated with such RA features as disease duration, rheumatoid factor positivity, ESR, C-reactive protein, DAS 28 or HAQ scores, prednisone doses (milligrams per day during the 3 previous months), current prednisone or DMARD intake (%S are not shown because they simply represent the reciprocal of IR). This lack of any correlation remained nonsignificant when data were adjusted for those classic risk factors associated with IR such as sex, age, BMI, or hypertension (adjusted model). Only C-reactive protein proved inversely related with IR (*P *= 0.05), although this was not maintained when adjusted for the classic IR risk factors.

As shown in Table 2, although classic IR triggers (BMI, waist circumference, and hypertension) were strongly associated with IR indices in RA patients, these classic risk factors made a dissimilar impact on IR indexes in patients and controls. In both RA and control groups, higher BMI, waist circumference, hypertension, and/or male sex were each generally associated with worse IR indexes (data not shown). However, the impact of BMI on the IR index in RA patients was more important than in controls (β coefficient 0.06 (0.03 to 0.10) versus 0.03 (0.01 to 0.05), *P *= 0.00). In contrast, the impact of waist circumference seemed more important in controls than in RA patients (β coef. 0.03 (0.01 to 0.04) versus 0.02 (0.01 to 03); *P *= 0.05), although statistic significance was not reached.

### β-Cell function markers and proinsulin-processing metabolites

Independent of prednisone intake, C-peptide levels were higher in non-steroid-taking patients compared with controls (*log*C-peptide, β coef. 0.20 nmol/L (0.02 to 0.38); *P *= 0.03). Insulin levels were also higher in noncorticoids RA patients than in those taking steroids. Conversely, neither insulin nor C-peptide correlated with prednisone dosing (milligrams per day) during the 3 previous months.

**Figure 1:**
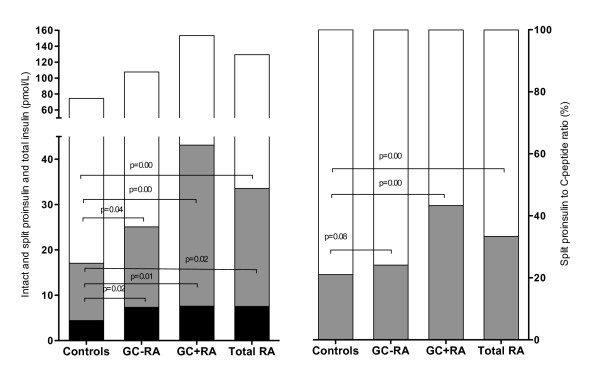
**Intact and split proinsulin in RA patients and controls**. Left graph (data plot on left axes) shows absolute values of intact proinsulin (black), split proinsulin (gray), and total insulin (white) in controls, non-glucocorticoids rheumatoid arthritis patients (GC-RA), rheumatoid arthritis patients taking glucocorticoids (GC+RA), and total rheumatoid arthritis patients (Total RA). Right graph (data plot on right axes) shows split proinsulin to C-peptide ratio (%) in the same groups. Significant *P *values are detailed.

Markers of β-cell dysfunction proved different between controls and patients (Figure 1). In this regard, split proinsulin, which expresses intact human proinsulin, as well as its major intermediate metabolites, des(31,32) proinsulin, and des(64,65) proinsulin, was higher in RA patients, (*log*Split insulin, β coef. 0.70 pmol/L (0.38 to 1.02); *P *= 0.00), even when adjusting for corticosteroid intake, sex, BMI, and age. The split proinsulin-to-C-peptide ratio was higher in RA patients undergoing corticosteroid therapy (β coef. 0.25 pmol/L (0.12 to 0.38); *P *= 0.03) and nearly reached significance in the comparison between noncorticoids patients and controls (β coef., 0.16 pmol/L (-0.02 to 0.34); *P *= 0.08); Figure 1 illustrates these differences.

Measurements of intact proinsulin levels showed similar results. Generally, patients expressed higher levels (β coef., 0.81 pmol/L (0.13 to 1.46); *P *= 0.02), and these data remained significant when adjusted for corticoids intake (Table 3). The intact proinsulin-to-C-peptide ratio was higher in patients compared with controls (β coef., 0.55 pmol/L (0.05 to 1.05); *P *= 0.03). When these data were adjusted for steroids consumption, statistical significance was not reached, although patients showed superior ratios.

**Table 3:** Associations of proinsulin-processing metabolites with RA features

	Β-coefficient (95% CI)			
	
Insulin and C-peptide (dependent variables)				
	***log*Insulin p*M***	***P***	***log*C-peptide**	***P***
	
RA versus controls (β 95% CI for RA, 101, and controls, 99)	**0.39 (0.14-0.64)**	**0.00**	**0.61 (0.32-0.91)**	**0.00**
GC-RA versus controls (β 95% CI for RA, 56, and controls, 99)	**0.14 (0.01-0.27)**	**0.04**	**0.20 (0.02-0.38)**	**0.03**
GC+RA versus controls (β 95% CI for RA, 55, and controls, 99)	**0.15 (0.06-0.25)**	**0.00**	**0.29 (0.17-0.40)**	**0.00**
RA patients features (β 95% CI for RA, 101)				
Disease duration	-0.79 (-3.32-1.74)	0.54	-0.01 (-0.02-0.00)	0.07
RF positivity	-10. 9 (-46.9-25.2)	0.99	0.03 (-0.06-0.12)	0.51
ESR	-0.50 (-1.49-0.50)	0.33	-0.00 (-0.01-0.00)	0.12
CRP	**-0.21 (-1.34-0.91)**	**0.03**	0.00 (-0.00-0.00)	0.65
Current prednisone	0.13 (-0.23-0.48)	0.48	**0.10 (0.01-0.18)**	**0.02**
Prednisone, mg/day/previous 3 months	-3.28 (-22.36-15.80)	0.25	-0.02 (-0.05-0.02)	0.16
HAQ	3.03 (-20.32-26.37)	0.63	-0.02 (-0.09-0.05)	0.69
DAS28	-0.21 (-10.89-10.49)	0.67	0.00 (-0.03-0.03)	0.99
Current nonbiologic DMARD	23.58 (-63.53-110.68)	0.79	0.05 (-0.13-0.23)	0.61
*log*Intact proinsulin (dependent variable)				

	**Intact proinsulin**	**p**	**Intact proinsulin/C-peptide ratio**	**p**
	
RA versus controls (β 95% CI for RA, 101, and controls, 99)	**0.81 (0.13-1.46)**	**0.02**	**0.55 (0.05-1.05)**	**0.03**
GC-RA versus controls (β 95% CI for RA, 56, and controls, 99)	**0.34 (0.05-0.62)**	**0.02**	1.65 (-0.06-3.35)	0.06
GC+RA versus controls (β 95% CI for RA, 55, and controls, 99)	**0.24 (0.05-0.43)**	**0.01**	0.14 (-0.04-0.33)	0.13
RA patients features (β 95% CI for RA, 101)				
Disease duration	0.03 (-0.46-0.51)	0.83	0.13 (-0.45-0.71)	0.96
RF positivity	3.4 (0.25-6.51)	0.11	3.45 (-0.34-7.25)	0.15
ESR	-0.03 (-0.12-0.06)	0.92	0.01 (-0.11-0.10)	0.62
CRP	0.02 (-0.07-0.12)	0.50	0.03 (-0.08-0.14)	0.47
Current prednisone	-0.07 (-0.73-0.59)	0.83	-1.40 (-5.30-2.50)	0.48
Prednisone. mg/day/previous 3 months	-0.11 (-1.86-1.65)	0.82	-0.05 (-1.83-1.73)	0.63
HAQ	0.62 (-2.13-3.37)	0.75	1.23 (-1.99-4.46)	0.85
DAS28	0.49 (-0.74-1.72)	0.65	0.39 (-1.03-1.82)	0.68
Current nonbiologic DMARD	2.93 (-7.52-13.34)	0.91	3.40 (-7.45-14.26)	0.96
*log*Split proinsulin (dependent variable)				

	**Split proinsulin**	***P***	**Split proinsulin/C-peptide ratio**	***P***
	
RA versus controls (β 95% CI for RA, 101, and controls, 99)	**0.70 (0.38-1.02)**	**0.00**	**0.57 (0.30-0.88)**	**0.00**
GC-RA versus controls (β 95% CI for RA, 56, and controls, 99)	**0.19 (0.00-0.36)**	**0.04**	0.16 (-0.02-0.34)	0.08
GC+RA versus controls (β 95% CI for RA, 55, and controls, 99)	**0.33 (0.20-0.45)**	**0.00**	**0.25 (0.12-0.38)**	**0.00**
RA patients features (β 95% CI for RA, 101)				
Disease duration	**-1.58 (-2.84-0.31)**	**0.01**	**-1.82 (-3.48-0.17)**	**0.02**
RF positivity	2.83 (-8.33-13.98)	0.60	5.11 (-8.54-18.76)	0.67
ESR	0.08 (-0.39-0.22)	0.60	-0.02 (-0.39-0.35)	0.93
CRP	0.16 (-0.18-0.51)	0.42	0.20 (-0.23-0.62)	0.33
Current prednisone	**0.57 (0.18-0.96)**	**0.00**	**0.41 (0.05-0.76)**	**0.03**
Prednisone. mg/day/previous 3 months	-1.04 (-6.99-4.91)	0.98	-0.14 (-6.50-6.22)	0.71
HAQ	-3.17 (-11.15-4.81)	0.11	-0.35 (-10.84-10.14)	0.43
DAS28	1.11 (-2.89-5.11)	0.82	-1.79 (-3.01-6.67)	0.67
Current nonbiologic DMARD	15.28 (-15.93-46.49)	0.83	15.43 (-17.36-48.23)	0.73

All β coefficients are adjusted for sex, age, and body mass index. All dependent variables are considered log transformed. Rheumatoid factor (RF) positivity, current prednisone and current nonbiologic DMARD are consider dichotomous variables. For abbreviations and units, see Table 1. Significant associations (*P *< 0.05) are depicted in bold

RA features like ESR, CRP, DAS28, HAQ, and rheumatoid factor positivity were not associated with β-cell marker or proinsulin-processing metabolite levels (Table 3). Only disease duration showed a significant negative association with split proinsulin and split proinsulin-to-C-peptide ratios. Similarly, current prednisone intake (as a dichotomous variable) was associated with a higher split insulin-to-C-peptide ratio.

A different relation between HOMA-IR and proinsulin metabolites was found in patients when compared with controls. Table 4 shows that controls generally expressed higher β coefficients of proinsulin metabolites over the same increase in the IR index. Thus, the impact of IR on the intact proinsulin-to-C-peptide ratio was higher in controls than in RA patients (6.23 (95% CI, 1.41 to 11.06) versus 0.43 (-0.86 to 1.71); *P *= 0.03); this association was not found in the split proinsulin/C-peptide ratio. When patients were divided into those taking corticoids and those who do not, no differences were found in the relation between HOMA-IR and split or intact proinsulin levels (Table 4).

**Table 4:** Different relation between HOMA-IR index and proinsulin metabolites in controls and patients.

	β coefficient (95% CI)			
	
	Intact proinsulin	Intact proinsulin/	Split proinsulin	Split proinsulin/
		C-peptide ratio		C-peptide ratio
HOMA-IR index				
Controls	4.02 (0.66-7.36)	6.23 (1.41-11.06)	10.95 (3.22-18.17)	14.84 (3.99-25.70)
Patients	0.97 (-0.11-2.06)	0.43 (-0.86-1.71)	6.98 (3.44-10.52)	5.83 (1.40-10.25)
*P *value^a^	0.36	**0.03**	0.47	0.23
RA on GC	1.28 (-0.42-2.98)	0.39 (-1.66-2.44)	6.94 (3.38-10.50)	5.37 (1.06-9.69)
RA non on GC	0.74 (-0.82-2.28)	5.55 (-1.26-2.36)	4.95 (-1.29-11.18)	3.70 (-4.13-11.72)
*P *value^b^	0.63	0.90	0.57	0.72

*P *value represents the comparison of β coefficient between controls and patients (^a^) and between RA on GC and RA not taking GCs (^b^) when interaction "HOMAIR × disease" is included in the regression model. HOMA-IR index is considered an independent variable, and proinsulin metabolites as dependent variables. Significant associations (*P *< 0.05) are depicted in bold

## Discussion

IR is characterized by hyperglycemia and relative impairment of insulin secretion. This condition has been described in RA patients, with its severity proving proportional to the degree of inflammatory activity [10,22,23], and this holds true for other inflammatory chronic diseases as well [24]. Understanding its pathogenesis is complicated because patients typically have varying degrees of both peripheral resistance and insulin deficiency. HOMA indices, although widely used, do not account for the differences observed in hepatic and peripheral insulin sensitivity, or for the variations seen in insulin secretion. For this reason, we decided to evaluate RA-related IR more accurately by measuring intact proinsulin, as well as its metabolites, as an expression of islet β-cell function. Our study suggests that the processing of proinsulin to insulin in β cells is impaired in nondiabetic noncorticoids RA patients. To our knowledge, this is the first study that has examined insulin resistance in RA with regard to these insulin-processing metabolites.

Insulin production in normal subjects involves cleavage of insulin from proinsulin; 10% to 15% of secreted insulin comprises proinsulin and/or its conversion intermediates. In contrast, the proportion of immunoreactive insulin that is proinsulin in type 2 diabetes increases considerably in the basal state. In our study, we detected an increase in intact and split proinsulin in RA patients with respect to healthy controls, a difference that was not corticoid mediated. The reasons for these increased levels of proinsulin and its related metabolites are likely multifactorial. It is possible that the IR recorded in the RA patients in our study may have arisen from inefficient proinsulin processing within the β-cell secretory granules or from the premature release of proinsulin as a result of increased demand for insulin due to systemic inflammation [25].

Elevations in the ratio of proinsulin to insulin, as well as in absolute proinsulin concentrations adjusted for fasting insulin, have been postulated as early markers of β-cell dysfunction [26]. However, proinsulin/C-peptide ratios have been shown to be a stronger predictor of diabetes [21]. These findings support the use of C-peptide as the best denominator for proinsulin ratios, because it more accurately reflects the degree of disproportional hyperproinsulinemia, which is in agreement with our findings that show higher and significant split proinsulin/C-peptide ratios in RA patients compared with controls. Some evidence links β-cell function and inflammation. Proinflammatory cytokines relevant to the pathogenesis of RA, such as interleukin-1β and tumor necrosis factor α, have been implicated in both the functional inhibition [27] and apoptosis induction [28] of islet β cells. These and other findings [29,30] suggest that inflammatory mediators might play a role in the β-cell dysfunction that we observed in RA patients.

It is difficult to separate the IR that occurs peripherally from that which arises because of, or after, β-cell damage. It remains unknown whether β-cell failure or a peripheral compensatory-like mechanism occurs first; however, once peripheral organs mount a resistance to insulin, pancreatic cell damage occurs. Interestingly, we found that when a similar increase in IR occurred, the intact proinsulin/C-peptide ratio was higher in controls than in RA patients. This could signify that the β-cell dysfunction observed in RA patients is at least in part independent of the severity of IR and allows the possibility that other factors, including inflammation, might be involved. Our data regarding IR levels in RA patients are in agreement with previous studies [4,22,23,31-33]. Surprisingly, in our series, however, we have not been able to find an association between RA disease activity scores, ESR, or CRP with IR, a finding that is not in agreement with the current evidence [10,22,23].

In the multiple regression analysis models, we found that abdominal obesity and BMI correlate with IR indices in both RA patients and controls. Abnormal body composition is a feature observed in RA, and the amount and distribution of fat and lean mass have important implications for IR [34]. Interestingly, according to our results, it seems that the impact of BMI on IR was higher in RA patients than in controls.

Because glucorticoids are an important factor affecting the development of hyperglycemia, and as they are usually prescribed to RA patients, corticosteroid intake and doses were examined in our study. In this regard, and in accordance with multiple regression analysis, we found that prednisone intake (as a dichotomous variable) or average prednisone dosing (milligrams per day during the 3 months prior) was not associated with IR indices in the intragroup of RA patients comparison, except for its relation with C-peptide and split proinsulin. Thus, our data are in keeping with previous reports contending that the use of high-dose glucocorticoids often results in hyperglycemia. However, it has been suggested that a low cumulative corticosteroid dose in RA patients may lead to improvement in β-cell function, as estimated by HOMA-%B [23]. For example, Toms *et al. *[35] did not find any association between corticosteroid use and the presence of metabolic syndrome in RA patients. In any case, the mechanisms associated with the development of metabolic syndrome related to corticosteroid use in RA need further elucidation.

We acknowledge that some limitations exist in our study. Both patients and controls exhibited high BMI, and thus IR indices and proinsulin levels might have been overestimated, particularly in controls. However, RA is a disease that is frequently associated with changes in BMI, and RA patients frequently have a high BMI. Second, as discussed earlier, corticosteroids exert pleiotropic metabolic effects that may be difficult to interpret in a chronic inflammatory disease setting, such as occurs in RA. This could therefore be likened to a double-edged sword, in that whereas corticosteroids have been associated with IR, at the same time, they confer beneficial antiinflammatory effects.

## Conclusions

In the present study, we observed that β-cell function is impaired in RA patients. Not only may the mechanisms leading to this condition be related to the disease itself, but they may also be different from those found in other IR-related conditions, such as diabetes mellitus and obesity.

## Abbreviations

BMI: body mass index; CRP: C-reactive protein; DAS28: Disease Activity Score; DMARD: disease-modifying antirheumatic drug; ELISA: enzyme-linked immunosorbent assay; ESR: erythrocyte sedimentation rate; GC: glucocorticoid; HAQ: Health Assessment Questionnaire; HDL: high-density lipoprotein; HOMA: Homeostatic Model Assessment; IR: insulin resistance; LDL: low-density lipoprotein; RA: rheumatoid arthritis; RF: rheumatoid factor; TNF: tumor necrosis factor.

## Competing interests

The authors declare that they have no competing interests.

## Authors' contributions

IFA and FDG designed the study and wrote the manuscript. JGD and LMV performed the laboratory studies. MAGG supported the design and revised the article. All authors read and approved the manuscript for publication.
